# Intrathecal idursulfase‐IT in children younger than 3 years with neuronopathic mucopolysaccharidosis II in a single‐arm, open‐label, phase 2/3 substudy and extension

**DOI:** 10.1002/jmd2.12443

**Published:** 2026-02-22

**Authors:** Joseph Muenzer, Barbara K. Burton, Paul Harmatz, Luis González Gutiérrez‐Solana, Matilde Ruiz‐Garcia, Simon A. Jones, Nathalie Guffon, Michal Inbar‐Feigenberg, Drago Bratkovic, Stewart Rust, Michael Hale, Yuna Wu, Karen S. Yee, David A. H. Whiteman, David Alexanderian

**Affiliations:** ^1^ University of North Carolina at Chapel Hill Chapel Hill North Carolina USA; ^2^ Ann & Robert H. Lurie Children's Hospital of Chicago Northwestern University Chicago Illinois USA; ^3^ UCSF Benioff Children's Hospital Oakland Oakland California USA; ^4^ Infant Jesus Children's Hospital Madrid Spain; ^5^ National Institute of Pediatrics Mexico City Mexico; ^6^ St Mary's Hospital Manchester University NHS Foundation Trust, University of Manchester Manchester UK; ^7^ Reference Center for Inherited Metabolic Diseases Hospices Civils de Lyon Lyon France; ^8^ University of Toronto Toronto Ontario Canada; ^9^ The Hospital for Sick Children Toronto Ontario Canada; ^10^ Women's and Children's Hospital North Adelaide South Australia Australia; ^11^ Manchester University NHS Foundation Trust Manchester UK; ^12^ Takeda Development Center Americas, Inc. Cambridge Massachusetts USA; ^13^ Hale Scientific Statistics, LLC Beaverton Oregon USA; ^14^ Takeda Development Center Americas, Inc. Lexington Massachusetts USA; ^15^ Present address: Alexion Pharmaceuticals, Inc., AstraZeneca Rare Disease Boston Massachusetts USA; ^16^ Present address: Merck Boston Massachusetts USA

**Keywords:** cognitive function, enzyme replacement therapy, idursulfase, infants, intrathecal, neuronopathic mucopolysaccharidosis II

## Abstract

Data from a phase 2/3, randomized, controlled, open‐label, multicenter trial in children with neuronopathic mucopolysaccharidosis II (MPS II; Hunter syndrome) older than 3 years suggested a benefit of intrathecal idursulfase‐IT on cognitive functioning in some patients. We describe a separate, parallel, open‐label, single‐arm, 52‐week substudy of the same trial (NCT02055118) that investigated idursulfase‐IT in children with MPS II younger than 3 years at enrollment and Bayley Scales of Infant and Toddler Development (BSID‐III) quotient 55–85. This report describes a prespecified analysis of nine patients (aged 1.4–3.0 years) who had received 3‐years' treatment with idursulfase‐IT. BSID‐III cognitive composite scores generally remained relatively stable over time. At the last available assessment, scores were “high average” (110; *n* = 1), “average” (100–90; *n* = 4), and “low average” (85–80; *n* = 4). Eight patients transitioned to the Differential Ability Scales (DAS‐II) after ages ≥42 months, and scores decreased for all patients when the instrument for assessing cognitive function changed. However, DAS‐II General Conceptual Ability scores were relatively stable for the remainder of the follow‐up. At the last available assessment, scores were “average” (106; *n* = 1), “low average” (85–80; *n* = 3), and “very low” (69–43; *n* = 4). Cerebrospinal fluid concentrations of total glycosaminoglycans were markedly reduced from baseline levels (mean [range] 1278 [429–2660] ng/mL) by week 16 and remained low thereafter. Data suggest early enzyme replacement therapy may stabilize cognitive development or slow the progression of cognitive impairment in young patients with neuronopathic MPS II.

**Synopsis**

Data from a prespecified substudy of a phase 2/3 trial suggest that treatment with intrathecal enzyme replacement therapy may have cognitive benefits in young patients (aged <3 years) with neuronopathic mucopolysaccharidosis II.

## 1 INTRODUCTION

Mucopolysaccharidosis II (MPS II; Hunter syndrome; OMIM 309900) is a rare, X‐linked, life‐limiting lysosomal storage disease characterized by deficient activity of iduronate‐2‐sulfatase (I2S) and subsequent accumulation of glycosaminoglycans (GAGs) in lysosomes throughout the body.^1^, ^2^ Approximately two‐thirds of patients with MPS II have a neuronopathic form of the disease, with central nervous system (CNS) involvement and cognitive impairment in addition to somatic symptoms.^1^, ^3^, ^4^, ^5^, ^6^ Although the age of onset of presenting signs and disease complications is variable, the median age at first symptom onset in the global Hunter Outcome Survey (HOS) registry was 1.5 years.^7^

Enzyme replacement therapy (ERT) with intravenous (IV) idursulfase (Elaprase, Takeda Pharmaceuticals USA, Inc., Lexington, MA, USA) or IV idursulfase beta (Hunterase, GC Biopharma, South Korea) is an approved treatment approach for patients with MPS II. Currently, IV idursulfase is the only treatment approved for patients with MPS II by the US Food and Drug Administration and the European Medicines Agency^8^, ^9^; IV idursulfase beta is approved in only some countries.^10^ Intracerebroventricular administration of idursulfase beta is also an approved treatment in Japan.^11^, ^12^ The patients in the original clinical trials of IV idursulfase were aged 5 years or older at baseline, and data from HOS have indicated a clinical benefit in terms of a reduction in liver volume and a reduction in urinary GAG levels in IV idursulfase‐treated patients between the ages of 16 months and 6 years. Given that IV idursulfase does not cross the blood–brain barrier at levels sufficient to have beneficial effects on cognitive impairment,^10^, ^13^ ERT with idursulfase using intrathecal administration (idursulfase‐IT) has been investigated as a treatment for the neurological manifestations of MPS II.^14^

A phase 2/3 study (HGT‐HIT‐094; NCT02055118) and its extension (SHP609‐302; NCT02412787) assessed the effect of idursulfase‐IT on cognitive function in children older than 3 years with neuronopathic MPS II.^15^, ^16^ The primary endpoint was not met; however, a post hoc analysis identified a clinically meaningful difference in change from baseline in cognitive scores at week 52 with idursulfase‐IT versus no idursulfase‐IT among those aged 3–6 years with missense variants in the gene encoding I2S (*IDS*).^15^

Given that patients younger than 3 years were not eligible for the pivotal phase 2/3 study, a separate, parallel, open‐label, single‐arm substudy was conducted to evaluate the safety and efficacy of idursulfase‐IT in this population. Here, we report safety and efficacy data from a prespecified interim analysis of these patients with MPS II and early MPS II‐related cognitive impairment younger than 3 years at enrollment, who received at least 36 months of idursulfase‐IT treatment in the substudy and the extension.

## 2 METHODS

### 2.1 Participants

Male patients with a documented diagnosis of MPS II and early MPS II‐related cognitive impairment were eligible for enrollment if they were younger than 3 years and had received treatment with IV idursulfase for at least 4 months immediately before screening. MPS II was defined as having deficient I2S activity (≤10% of the lower limit of normal) as measured in plasma, fibroblasts, or leukocytes, together with either: (a) a documented *IDS* variant that leaves the fragile X mental retardation genes (*FMR1* and *FMR2*) intact; or (b) normal enzyme activity levels of one other sulfatase as measured in plasma, fibroblasts, or leukocytes. MPS II‐related cognitive impairment was defined as having a development quotient of 55–85 on the Bayley Scales of Infant and Toddler Development, Third Edition (BSID‐III).^17^

Exclusion criteria were the same as those for the pivotal phase 2/3 study, and have been detailed in full previously.^15^

### 2.2 Study design

This open‐label, single‐arm substudy was conducted in parallel to the pivotal phase 2/3 study. Details of study sites, dates, informed consent, and ethical approval have been previously published.^15^, ^16^ Enrolled patients received idursulfase‐IT every 28 days, in addition to weekly IV idursulfase, for 52 weeks in the substudy. On completion, patients were eligible to participate in an ongoing, open‐label, non‐randomized extension study, in which they would continue to receive idursulfase‐IT every 28 days and weekly IV idursulfase.

Idursulfase‐IT was administered intrathecally via an implantable IT drug delivery device (IDDD) (SOPH‐A‐PORT Mini S device) or by lumbar puncture every 28 days. Based on reference brain weight,^18^ the dose of idursulfase‐IT was 7.5 mg for patients aged 8–30 months; and 10 mg for patients older than 30 months. In the substudy, the IV infusion was administered a minimum of 48 h after IT administration of idursulfase‐IT; in the extension study, same‐day administration was permitted.

### 2.3 Endpoints and assessments

The substudy aimed to examine the effects of idursulfase‐IT treatment on safety and efficacy outcomes in boys younger than 3 years with MPS II and early cognitive impairment. Patients in the substudy followed a similar schedule of assessments and study visits to those previously reported for the pivotal phase 2/3 study and extension.^15^, ^16^

In the substudy, cognitive function was initially assessed using the BSID‐III cognitive scale.^17^ When patients reached at least 42 months of age, assessments were performed using the Differential Ability Scales, Second Edition (DAS‐II [early years battery, designed for children aged 2 years 6 months to 6 years 11 months])^19^ General Conceptual Ability (GCA) scores, if the DAS‐II was deemed appropriate in each individual patient. For the three main DAS‐II subtests (verbal comprehension, naming vocabulary, and pattern construction), the first item set was administered initially and if the patient could not complete these, the BSID‐III was used.

Adaptive behavior was assessed using the Vineland Adaptive Behavior Scales, Second Edition (VABS‐II)^20^ Expanded Interview Form. The VABS‐II Adaptive Behavior Composite (ABC) score provides an overall measure of adaptive behavior ability, and is a composite score of domains for communication, daily living, socialization, and motor skills. The VABS‐II ABC and VABS‐II domains have normative scores from birth to 90 years of age with a mean of 100 and a standard deviation of 15.

BSID‐III, DAS‐II, and VABS‐II assessments were made at baseline and at weeks 16, 28, 40, and 52 in the substudy, and every 6 months thereafter in the extension study.

Safety assessments included collection of adverse events (AEs), clinical laboratory testing, physical and neurological examination, vital signs, and evaluation of anti‐idursulfase antibodies in the cerebrospinal fluid (CSF) and serum. Pharmacodynamic endpoints included the change from baseline in total GAG and heparan sulfate (HS) levels in the CSF; levels were evaluated at baseline and at weeks 4, 16, 28, 40, and 52 in the substudy, and every 3 months thereafter in the extension study. CSF samples were collected and assessed as previously described.^15^, ^16^

### 2.4 Statistical analysis

There was no target sample size; all patients meeting eligibility criteria were enrolled until enrollment for the pivotal trial was complete. A prespecified interim analysis was performed after 3 years, when all patients had received at least 36 months of idursulfase‐IT treatment across the substudy and the extension (completing the month 37 [week 148] visit). BSID‐III cognitive and language composite scores, DAS‐II GCA and verbal cluster scores, and VABS‐II ABC and domain scores were summarized for individual patients over the course of the substudy and extension. Safety and efficacy data were summarized descriptively or listed for individual patients.

## 3 RESULTS

### 3.1 Patients

In total, nine patients were enrolled into the substudy, all of whom were continuing to receive treatment in the extension at the 3‐year data cutoff (Table 1). Patient age at study baseline ranged from 1.4 to 3.0 years, and median (range) age at diagnosis of MPS II was 1.6 (0.0–1.9) years. Four patients (diagnosis at birth, and aged 5, 7, and 9 months; symptom onset aged 5–8 months) had a family history of MPS II (one with a cousin with MPS II, two with brothers, and one with two brothers). The *IDS* variant classifications for the nine patients were: four missense, three deletions, one splicing, and one nonsense. BSID‐III cognitive composite scores at screening ranged from 75 to 90: patients 3 and 8 had “average” scores (both 90), patients 1, 4, 5, 6, 7, and 9 had “low average” scores (either 80 or 85), and patient 2 had a “borderline” score (75). BSID‐III language composite scores were more widely distributed (range: 59–91) at screening (Table 1).

**TABLE 1:** Baseline characteristics.

Characteristics	Patient number								
	1	2	3	4	5	6	7	8	9
Age, years	2.5	2.6	2.4	3.0	2.7	1.4	2.6	3.0	2.2
Genotype	c.419‐2A>G	Deletion of exons 1–8 of *IDS*	c.257C>T	c.257C>T	c.671G>A	Deletion of exons 8 and 9 of *IDS*	Deletion of exons 1–9 of *IDS*	c.1506G>C	c.514C>T
Genotype classification	Splicing	Deletion	Missense	Missense	Missense	Deletion	Deletion	Missense	Nonsense
Amino acid substitution	–	–	p.P86L	p.P86L	p.G224E	–	–	p.W502C	p.R172X
Family history of MPS II	Yes	Yes	Yes	Yes	No	No	No	No	No
BSID‐III cognitive development quotient	75.1	69.3	84.8	64.5	73.2	72.3	79.7	81.4	83.8
BSID‐III cognitive composite score	85	75	90	80	85	80	85	90	85
BSID‐III language composite score	91	74	79	79	59	89	79	86	62

*Note*: English was not the primary language of patients 8 and 9, which limits the interpretation of the BSID‐III language scale in these children.

Abbreviations: BSID‐III, Bayley Scales of Infant and Toddler Development, Third Edition; *IDS*, iduronate‐2‐sulfatase gene; MPS II, mucopolysaccharidosis II.

Seven patients received idursulfase‐IT via both IDDD and lumbar puncture, one via IDDD only, and one by lumbar puncture only (Table S1). Overall, 97.8% of the planned doses were administered over the 36‐month study period; of the nine patients, five received all scheduled monthly doses of idursulfase, two missed one dose, and two missed four doses. The median (range) number of doses of idursulfase‐IT overall was 45 (36–52): 33.5 (16–51) via IDDD and 9.5 (1–46) by lumbar puncture.

### 3.2 Cognitive function

**FIGURE 1:**
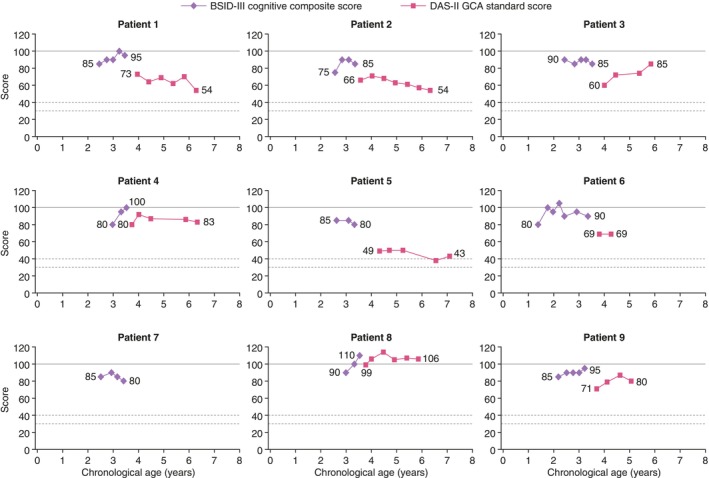
BSID‐III cognitive composite scores and DAS‐II GCA scores by age in patients treated with idursulfase‐IT. The BSID‐III cognitive composite score and DAS‐II GCA standard score have a mean of 100 (solid line). Cognitive function was initially assessed using the BSID‐III cognitive scale. When patients reached at least 42 months of age, assessments were performed using the DAS‐II, if the DAS‐II was deemed appropriate in each individual patient. Dashed horizontal lines show DAS‐II GCA scores of 30 and 40. The DAS‐II GCA score has a lower limit (floor) of 30; to account for variability, 10 points were added to this threshold, such that any patient with a score below 30 or 40 was considered to have reached the floor. BSID‐III, Bayley Scales of Infant and Toddler Development, Third Edition; DAS‐II, Differential Abilities Scale, Second Edition; GCA, General Conceptual Ability; IT, intrathecal.

Following enrollment into the substudy and treatment with idursulfase‐IT in addition to IV idursulfase, BSID‐III cognitive composite scores generally remained relatively stable over time (Figure 1). At the last available BSID‐III assessment, patient 8 had a “high average” score (110), patients 1, 4, 6, and 9 had “average” scores (95, 100, 90, and 95, respectively), and patients 2, 3, 5, and 7 had “low average” scores (85, 85, 80, and 80, respectively).

Scores decreased for all patients when the instrument for assessing cognitive function changed from the BSID‐III to the DAS‐II (Figure 1). At the first available DAS‐II assessment, patient 8 had an “average” GCA score (99), patient 4 had a “low average” GCA score (80), patients 1 and 9 had “low” GCA scores (73 and 71), and patients 2, 3, 5, and 6 had “very low” GCA scores (66, 60, 49, and 69, respectively). Patient 7 was not assessed with DAS‐II, and BSID‐III cognitive composite scores were not calculated for this patient during the extension as the patient was over the age for test norms. Among the patients with two or more DAS assessments, 5 out of 8 either had stabilized or improved cognitive function over more than 2 years of assessments (Figure 1). At the last available DAS‐II assessment before the interim analysis, patient 8 had an “average” GCA score (106), patients 3, 4, and 9 had “low average” GCA scores (85, 83, and 80), and patients 1, 2, 5, and 6 had “very low” GCA scores (54, 54, 43, and 69, respectively).

### 3.3 Language/verbal skills

**FIGURE 2:**
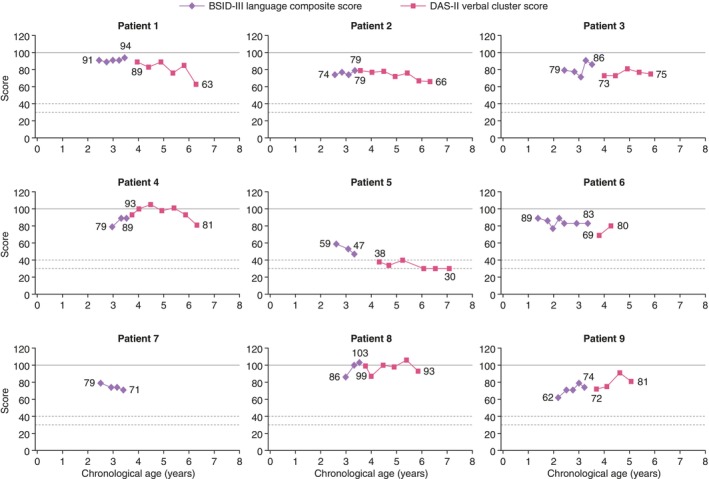
BSID‐III language composite scores and DAS‐II verbal cluster scores by age in patients treated with idursulfase‐IT. The BSID‐III language composite score and DAS‐II GCA verbal cluster score have a mean of 100 (solid line). Dashed horizontal lines show cluster/composite scores of 30 and 40. English was not the primary language of patients 8 and 9, which limits the interpretation of the BSID‐III language composite scores in these children. BSID‐III, Bayley Scales of Infant and Toddler Development, Third Edition; DAS‐II, Differential Abilities Scale, Second Edition; GCA, General Conceptual Ability; IT, intrathecal.

BSID‐III language composite scores generally remained stable in most patients over time (Figure 2), although English was not the primary language of patients 8 and 9, which limits the interpretation of the BSID‐III language scale for these children. At the last available BSID‐III language assessment, patients 1 and 8 had “average” scores (94 and 103, respectively), patients 3, 4, and 6 had “low average” scores (86, 89, and 83, respectively), patients 2, 7, and 9 had “low” scores (79, 71, and 74, respectively), and patient 5 had a “very low” score (47).

DAS‐II verbal cluster scores appeared relatively stable for the remainder of the 3 years of treatment (Figure 2). At the last available assessment of DAS‐II verbal cluster scores before the interim analysis, patient 8 had an “average” score (93), patients 4, 6, and 9 had “low average” scores (81, 80, and 81), patients 1, 2, and 3 had “low” scores (63, 66, and 75, respectively), and patient 5 had a “very low” score (30).

### 3.4 Adaptive behavior

**FIGURE 3:**
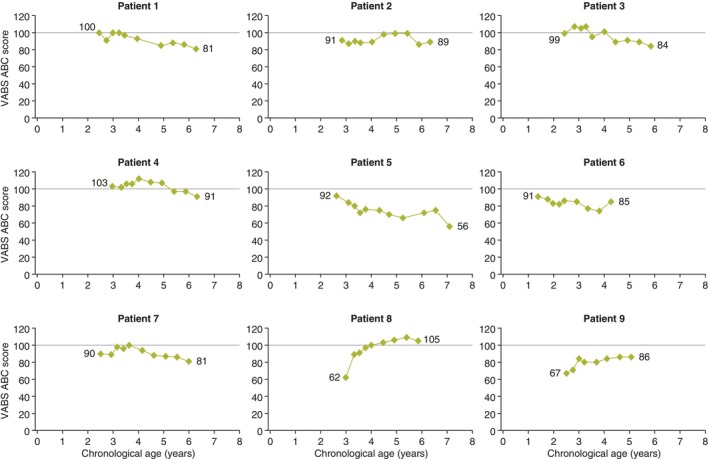
VABS‐II ABC scores by age in patients treated with idursulfase‐IT. The VABS‐II ABC score provides an overall measure of adaptive behavior ability, and is a composite score of domains for communication, daily living, socialization, and motor skills. The VABS‐II ABC score has a mean of 100 (solid line). ABC, Adaptive Behavior Composite; IT, intrathecal; VABS‐II, Vineland Adaptive Behavior Scales, Second Edition.

VABS‐II ABC scores over time are shown in Figure 3. Where available (baseline scores were not available for patients 2 and 9), baseline VABS‐II ABC scores ranged from 90 to 103, except in patient 8, for whom the score was notably lower (62). For most patients, VABS‐II ABC scores remained relatively stable; however, patient 5 (who had low BSID‐III language and DAS‐II verbal cluster scores) had a steady decrease in VABS‐II ABC score. In patient 8, the VABS‐II ABC score increased to 100 by week 52 and remained at 100 or higher thereafter. VABS‐II domain scores for communication, daily living skill, socialization, and motor skill over time to month 37 are shown in Figure S1.

### 3.5 CSF total GAG and HS

**FIGURE 4:**
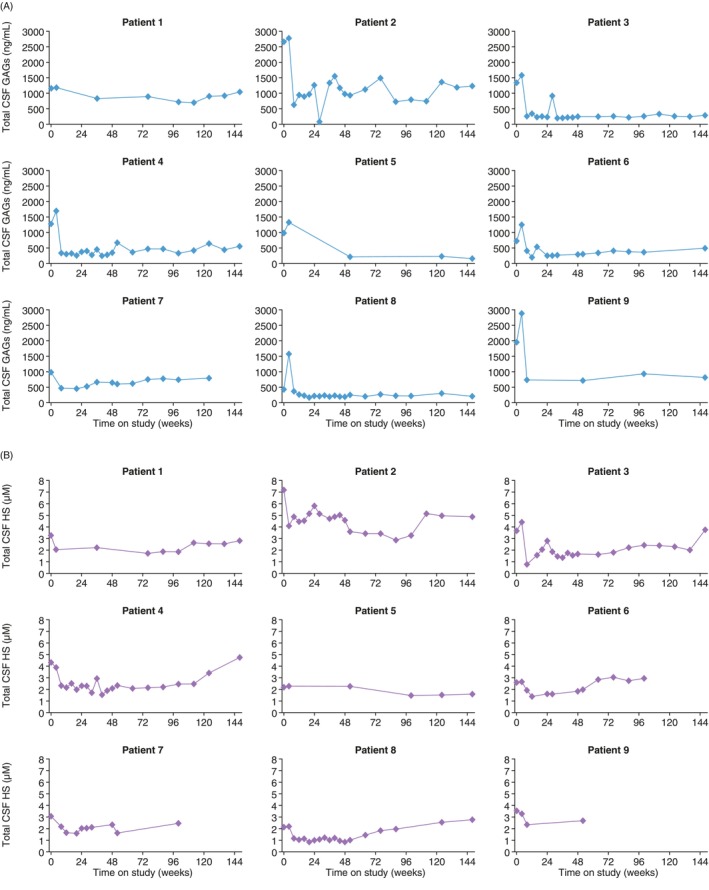
CSF (A) total GAG and (B) HS in patients treated with idursulfase‐IT for 3 years. Samples of CSF were collected via the IDDD. Total CSF GAG concentrations were quantified by the study sponsor using a thrombin activity assay. Levels of HS in CSF were determined using a validated liquid chromatography–mass spectrometry/mass spectrometry assay, which measured N‐butylaniline‐derivatized disaccharides from the digestion of CSF HS by heparinases; the lower limit of quantification was 0.100 μM. HS assays were conducted by a sponsor‐designated contract research organization (Labcorp Drug Development, Burlington, NC, USA). Reported mean CSF GAG levels in individuals without MPS II are 50–70 ng/mL, depending on age, and maximums are below approximately 200 ng/mL.^41^ Reported mean CSF HS levels in patients without MPS II are 0.37 μM.^42^ CSF HS data for patient 9 were unavailable after week 52 (reason unknown). CSF, cerebrospinal fluid; GAG, glycosaminoglycan; HS, heparan sulfate; IDDD, implantable IT drug delivery device; IT, intrathecal; MPS II, mucopolysaccharidosis II.

The mean (range) baseline total CSF GAG concentration was 1278 (429–2660) ng/mL and baseline HS concentration was 3.55 (2.12–7.18) μM. In general, CSF total GAG concentrations were markedly reduced from baseline by week 16 and remained low thereafter through month 37 (Figure 4A). CSF HS concentrations were initially reduced after initiation of idursulfase‐IT, but there was a trend for concentrations to return toward baseline values over time (Figure 4B).

### 3.6 Safety and anti‐idursulfase antibodies

In this population of patients aged 1.4–3.0 years at enrollment, idursulfase‐IT was generally well tolerated, with no discontinuations and no deaths during the follow‐up. An overview of the reported AEs, serious AEs (SAEs), and other safety assessments is shown in Table S2. Device‐related failures or infections were common, as were pyrexia, diarrhea, and vomiting. Most events were considered mild or moderate and unrelated to treatment. All SAEs were mild or moderate, except one case of device‐related sepsis and CNS infection considered probably related to the IDDD.

At baseline, seven patients (77.8%) tested positive for serum anti‐idursulfase antibodies, with six of these patients (85.7%) testing positive for neutralizing antibodies (Table S3). Six patients (66.7%) tested positive for CSF anti‐idursulfase antibodies at baseline, although neutralizing antibodies were not found in the CSF (Table S3). After baseline, all patients tested positive for neutralizing antibodies in the serum, although one patient tested positive on only one occasion. For neutralizing antibodies in the CSF, patients 3, 4, and 8 did not test positive at any point, and only patients 1 and 2 tested positive on more than one occasion; the other four patients (patients 5, 6, 7 and 9) tested positive for CSF neutralizing antibodies at week 4 only. Serum and CSF anti‐idursulfase antibody titers are shown in Figure S2.

## 4 DISCUSSION

This open‐label, single‐arm substudy enabled patients younger than 3 years with neuronopathic MPS II to receive treatment with idursulfase‐IT. All nine patients who were enrolled completed the substudy and entered the extension study, allowing long‐term evaluation of idursulfase‐IT treatment over at least 3 years. In this population, idursulfase‐IT was generally well tolerated, and cognitive scores remained relatively stable, as determined initially using BSID‐III and subsequently DAS‐II in all but one patient.

This study employed established tools for the assessment of cognitive ability. The BSID‐III is considered to be the gold standard tool for assessing cognitive function in infants and toddlers.^21^ It is appropriate for use in infants, toddlers, and young children between the ages of 1 and 42 months,^22^ so it was not used throughout the study as the children grew older than 42 months. The DAS‐II was developed to assess cognitive abilities in children from the age of 2 years 6 months up to 18 years of age and was considered an appropriate cognitive ability assessment tool for children with neuronopathic MPS II at the time of the study implementation.^23^, ^24^ Adaptive behavior scales measure performance of activities as reported by the parent or caregiver and help to contextualize cognitive function scores; the VABS‐II is based on interviews with an outside observer, such as a parent or a caregiver,^20^ and is the recommended measurement of adaptive behavior for all patients with MPS II.^21^

Existing data on the natural history of MPS II suggest that the trajectory of neuronopathic disease varies considerably with respect to the age at which slowing of cognitive development and decline occur in patients.^25^ Cognitive development may be normal during the first few years of life, followed by a plateau in development in early childhood, and then progressive neurocognitive decline.^25^, ^26^, ^27^ Caution should be taken with regard to the reported stabilization of cognitive function following treatment, because it would depend on when in the developmental trajectory the measurements were taken. However, stabilization, or a slower or delayed decline, can be viewed as a positive and clinically meaningful outcome for patients with progressive lysosome storage diseases.^28^ The generally stable cognitive function observed with idursulfase‐IT was consistent with the findings from a post hoc analysis of data from the pivotal phase 2/3 study, which showed a clinically meaningful difference in change from baseline in cognitive scores in a subgroup of young patients (between 3 and 6 years of age) with MPS II and missense *IDS* variants.^15^ Longitudinally, the DAS‐II GCA score can be considered stable within two standard errors (approximately 10 points).^29^ Although there was generally a decrease in observed scores when transitioning from the BSID‐III to the DAS‐II, only one patient had a clear decrease of more than 10 points, indicating that the cognitive functioning of most patients in this study developed at similar rates to same‐aged unaffected children. One patient (patient 5) was nonverbal, had DAS‐II GCA scores that approached the assessment floor, and DAS‐II verbal cluster scores of 40 or lower from the age of approximately 4.5 years. The BSID‐III cognitive scores for this patient had been 80–85 during the study, with language composite scores decreasing from 59 to 47 between the ages of approximately 2.5–3.5 years, although he continued to gain language skills.

This substudy provided an opportunity to gather data from younger patients than those enrolled and randomized in the pivotal clinical trial, although the small number of patients enrolled in this study precludes formal statistical analysis of associations with genotype. *IDS* variants that cause large structural alterations, such as complete/large deletions, recombinations, frameshift variants, and nonsense variants, have been associated with the neuronopathic MPS II phenotype. By contrast, patients with missense variants show a high degree of heterogeneity in their presenting phenotype.^30^, ^31^, ^32^ Four of the nine participants (patients 3, 4, 5, and 8) had missense *IDS* variants and, at the time of enrollment, cognitive functioning was not clearly different from that in patients with other variant types. Considering the molecular heterogeneity of missense variants, this observation is neither surprising nor incompatible with the possibility that milder *IDS* variants in MPS II that lead to higher residual enzyme activity may contribute to different sensitivity to treatment. These patients may also be less likely to develop antibodies to idursulfase which, in turn, may also affect treatment effectiveness.^33^ It is important to note the stabilization or lack of obvious disease progression observed over 3 years of follow‐up in the young patients with *IDS* deletions (patients 2, 6, and 7), despite rapid cognitive decline having been consistently observed in patients with similar *IDS* variants without idursulfase‐IT treatment. Nonetheless, establishing genotype–phenotype correlations in MPS II is challenging and requires larger patient populations with longitudinal follow‐up.^25^, ^26^

Natural history studies have generally found similar trajectories for cognitive abilities and adaptive functions in patients with other mucopolysaccharidoses,^21^ but data in infants and toddlers with MPS II are lacking. In this study, all nine patients had adaptive functioning scores within the “adequate” range (typically considered for scores 86–114)^20^ at first assessment (undertaken between the ages of 1.4 and 3.0 years). After at least 3 years of treatment with idursulfase‐IT, all patients maintained VABS‐II ABC scores in the “adequate” range, except one patient with a score at the upper end of the “moderately low” range, and one patient with a “low” score. These findings appear promising, although the lack of comparative data in a similarly young population with MPS II limits interpretation.

Although total CSF GAG levels demonstrated sustained reductions over 3 years of idursulfase‐IT treatment, there was no clear decrease in CSF HS levels over the long‐term follow‐up, with levels trending back to baseline, mostly after the second year of treatment; this was also observed in the main extension.^16^ The trend for partial reversal of initial reductions of CSF HS levels, which continued with ongoing treatment, was thought to be possibly dose‐related and a possible explanation as to why the cognitive results were not more pronounced in the overall study.^16^ Across the nine patients in the substudy, it was difficult to determine any clear associations between treatment benefit and CSF GAG or HS levels. For example, patient 2 (with an *IDS* deletion) had the highest CSF HS levels at baseline and throughout the study but had small numerical increases in VABS‐II scores, BSID‐III cognitive and language composite scores, and DAS‐II verbal cluster scores, and a decrease of only five points in DAS‐II GCA scores over 3 years. Interestingly, patient 2 was one of the two patients who had neutralizing antibodies in the CSF on more than one occasion in the substudy. However, CSF HS levels did not appear to be different in patients with neutralizing antibodies, compared with those without neutralizing antibodies.

Overall, idursulfase‐IT was generally well tolerated in the substudy population, with no deaths or discontinuations due to AEs over the study period of at least 3 years. AEs were generally mild or moderate and unremarkable relative to the safety and tolerability profile of idursulfase‐IT reported in the pivotal phase 2/3 trial and extension.^15^, ^16^ All but one child received idursulfase‐IT via the IDDD, although administration via lumbar puncture was necessary on occasion.

Evidence from studies of the effects of IV idursulfase on somatic symptoms of MPS II has been strongly supportive of early initiation of ERT, before pathological changes are established.^34^ For example, a case series of eight patients with MPS II who started treatment with IV idursulfase between the ages of 10 days and 6.5 months revealed improvements or stabilization of some somatic manifestations during treatment, with anecdotal reports of improved clinical course in patients with earlier versus later treatment initiation.^35^ Similarly, a study of two siblings provided support for the presymptomatic initiation of ERT for the prevention or slowed progression of somatic features of the disease.^36^ It might be expected that interventional treatments for neuronopathic MPS II may also need to be initiated at a young age to optimize the impact on the disease course. Data from the pivotal phase 2/3 study of idursulfase‐IT supported this hypothesis, with a post hoc analysis revealing that patients with missense *IDS* variants initiating idursulfase‐IT treatment before the age of 6 years were the most likely to experience benefits in cognitive outcomes.^15^ With the adoption of newborn screening programs,^37^, ^38^, ^39^ our understanding of MPS II and its phenotypes will advance over the coming years^40^; hence, it will be even more critical that we continue to investigate treatment outcomes in young children. Although not tested statistically, the findings of this substudy were consistent with the principle of early treatment in neuronopathic MPS II. However, after many years of extensive review and regulatory discussions, the idursulfase‐IT data overall were found to be insufficient to meet the evidentiary standard to support regulatory filings.

Given that MPS II is a rare disease,^35^, ^36^ this study of a small subpopulation of patients with MPS II is limited by the low number of patients. In addition, the study design did not incorporate a control group or statistical comparisons. Interpretation of the data is also restricted by the lack of natural history information for patients with MPS II in this age group, as well as by the complexity of available data. The switch in instruments for the assessment of cognitive development due to the age of the patients during the long‐term follow‐up affected the continuity of the data, despite being an approach that has been used in a previous study.^23^

## 5 CONCLUSIONS

These data suggest that early idursulfase‐IT treatment may stabilize cognitive development or slow the progression of cognitive impairment in very young patients with neuronopathic MPS II. Idursulfase‐IT will continue to be made available to patients who were enrolled in the ongoing open‐label extension studies until an alternative approved treatment option is available to address the cognitive symptoms.

## AUTHOR CONTRIBUTIONS

JM, BKB, PH, LGG‐S, MR‐G, SAJ, NG, MI‐F, DB, and SR were involved in the acquisition of the data. MH, YW, KSY, DAHW, and DA were involved in the initial conception or design of the study. All authors contributed to the interpretation of the data, to the drafting of the manuscript, provided critical review during revisions, and approved the final manuscript for submission.

## FUNDING INFORMATION

These studies were sponsored and funded by Shire (a Takeda company). Under the direction of the authors, medical writing support was provided by Sarah Feeny BMedSci of Oxford PharmaGenesis, Oxford, UK, and was funded by Takeda Development Center Americas, Inc.

## CONFLICT OF INTEREST STATEMENT

JM has received consulting fees from and has participated on data safety monitoring boards or advisory boards for Denali Therapeutics, JCR Pharmaceuticals, REGENXBIO, Sanofi Genzyme, and Takeda; is a Principal Investigator for a post‐trial access program for intrathecal ERT for the neuronopathic form of MPS II (sponsored by Takeda), a phase 1/2 gene editing trial for adults with MPS II (sponsored by Sangamo Therapeutics), and phase 1/2 and phase 2/3 trials of IV ERT for MPS II (sponsored by Denali Therapeutics).

BKB has received consulting fees from Aeglea, Agios, Alexion, AstraZeneca Rare Disease, Alltrna, BioMarin Pharmaceutical, Chiesi Farmaceutici, Horizon Therapeutics, JCR Pharmaceuticals, Jnana Therapeutics, Moderna, Orchard Therapeutics, Passage Bio, PTC Therapeutics, REGENXBIO, Sanofi Genzyme, Takeda, Travere, and Ultragenyx; has received payment or honoraria for lectures, presentations, speakers bureaus, manuscript writing, or educational events from Alexion, BioMarin Pharmaceutical, Chiesi Farmaceutici, Horizon Therapeutics, Sanofi Genzyme, Takeda, and Ultragenyx; has participated on data safety monitoring boards or advisory boards for BioMarin Pharmaceutical, Freeline, JCR Pharmaceuticals, Moderna, and Takeda; has performed contracted research for Takeda and has been involved in company‐sponsored clinical trials with BioMarin Pharmaceutical, Denali Therapeutics, Homology Medicines, JCR Pharmaceuticals, Jnana Therapeutics, Sangamo Therapeutics, Synlogic, Takeda, and Ultragenyx.

PH has received grants or contracts from BioMarin Pharmaceutical, Denali Therapeutics, Grace Science, Takeda, QED, Adrenas Therapeutics, Sangamo, Prevail/Lilly, Ascendis, ASPA, Idorsia, JCR Pharmaceuticals, Orphazyme, REGENXBIO, Sanofi Genzye, Calcilytics, Immusoft, Allievex, Amicus Therapeutics, Azafaros, and Homology. He has received consulting fees from Aeglea, BioTherapeutics, Audentes, AVROBIO, Capsida Biotherapeutics, Chiesi, Edigene, Grace Science, Inventiva Pharma, Neurogene, Novel Pharma, Orchard Therapeutics, Rallybio, Renoviron, Saliogen, and Sanofi Genzyme; and has received payment or honoraria for lectures, presentations, speakers bureaus, manuscript writing, or educational events from BioMarin Pharmaceutical.

LGG‐S has received consulting fees from BioMarin Pharmaceutical, Sanofi Genzyme, Takeda, and Ultragenyx Pharmaceutical; has received payment or honoraria for lectures, presentations, speakers bureaus, manuscript writing, or educational events from BioMarin Pharmaceutical, Sanofi Genzyme, Takeda, and Ultragenyx Pharmaceutical and has received research support from Takeda.

MR‐G has received consulting fees, payment, or honoraria for lectures, presentations, speakers bureaus, manuscript writing, or educational events and research support from Takeda.

SAJ has received grants or contracts from Orchard Therapeutics (SRAs and consulting plus investigator); has received consulting fees and payment or honoraria for lectures, presentations, speakers bureaus, manuscript writing, or educational events from Alexion Pharmaceuticals, AVROBIO, BioMarin Pharmaceutical, Denali Therapeutics, Orchard Therapeutics, REGENXBIO, Sanofi Genzyme, Takeda, and Ultragenyx Pharmaceutical. He has received support for attending meetings and/or travel from Sanofi and has received research support from Takeda.

NG has received research support from BioMarin Pharmaceutical, Chiesi, Sanofi Genzyme, Takeda, and Ultragenyx Pharmaceutical.

MI‐F has performed contracted research for Takeda, Denali Therapeutics, Sanofi Genzyme, Ultragenyx Pharmaceutical, Passage Bio, Aeglea Biotherapeutics, PTC Therapeutics, and Vtesse (Mallinckrodt Pharmaceuticals), and received the Canadian Institute of Health Research Strategy for Patient Oriented Research Innovative Clinical Trial Multi‐Year Grant; has received consulting fees from Sanofi Genzyme, Takeda, and Simon‐Kucher; has received payment or honoraria for lectures, presentations, speakers bureaus, manuscript writing, or educational events from Horizon Therapeutics; has participated on a data safety monitoring board or advisory board for Alexion Pharmaceuticals, Cyclo Therapeutics, Horizon Therapeutics, Recordati Rare Diseases, Sanofi Genzyme, Takeda, and Ultragenyx Pharmaceutical; is a member of the medical advisory board for the Canadian MPS Society and Allied Diseases and is a member of the Garrod Association Guideline Committee.

DB has received research support from Takeda.

SR has received consulting fees from AVROBIO, BioMarin Pharmaceutical, Chiesi Farmaceutici, Takeda, and Ultragenyx; has received payment or honoraria for lectures, presentations, speakers bureaus, manuscript writing or educational events, payment for expert testimony, and support for attending meetings and/or travel from AVROBIO, BioMarin Pharmaceutical, Chiesi Farmaceutici, and Takeda.

MH was an employee of Takeda Development Center Americas, Inc. at the time of this study; and has stock or stock options for Takeda Pharmaceuticals Company Limited.

YW is an employee of Takeda Development Center Americas, Inc.; and has stock or stock options for Takeda Pharmaceuticals Company Limited.

KSY was an employee of Takeda Development Center Americas, Inc. at the time of this study and of the writing of the manuscript (current affiliation is Alexion Pharmaceuticals, Inc., AstraZeneca Rare Disease, Boston, MA, USA) and has stock or stock options for Takeda Pharmaceuticals Company Limited and AstraZeneca.

DAHW was an employee of Takeda Development Center Americas, Inc.; and was a stockholder of Takeda Pharmaceuticals Company Limited at the time of the study.

DA was an employee of Takeda Development Center Americas, Inc. at the time of this study and of the writing of the manuscript (current affiliation is Merck, Boston, MA, USA) and has stock or stock options for Takeda Pharmaceuticals Company Limited.

## ETHICS STATEMENT

The studies were conducted in accordance with the Declaration of Helsinki and written informed consent was obtained from the parent(s) or legally authorized guardian(s); assent from the patient was also acquired, if applicable.

## Supporting information


**Table S1.** Idursulfase‐IT treatment administration by intrathecal drug delivery device (D) or lumbar puncture (LP).
**Table S2.** Summary of TEAEs in patients treated with idursulfase‐IT.
**Table S3.** Idursulfase anti‐drug antibody and neutralizing antibodies in serum and CSF.
**Figure S1.** VABS‐II communication domain (A), daily living skills (B), socialization (C), and motor skills (D) scores by age in patients treated with idursulfase‐IT.
**Figure S2.** Serum (A) and CSF (B) anti‐idursulfase antibody titers in patients treated with idursulfase‐IT.

## Data Availability

The data sets, including the redacted study protocol, redacted statistical analysis plan, and individual participants' data supporting the results reported in this article, will be made available within 3 months from initial request to researchers who provide a methodologically sound proposal. The data will be provided after de‐identification in compliance with applicable privacy laws, data protection, and requirements for consent and anonymization.
