# Supplementary material for: Intrathecal idursulfase‐IT in children younger than 3 years with neuronopathic mucopolysaccharidosis II in a single‐arm, open‐label, phase 2/3 substudy and extension
Source: JIMD Rep. 2026 Feb 22;67(2):e12443. doi: 10.1002/jmd2.12443 (PMC12928010; doi:10.1002/jmd2.12443)

# Supplementary materials Intrathecal idursulfase‑IT in children younger than 3 years with neuronopathic mucopolysaccharidosis II: single-arm, open-label phase 2/3 substudy and extension

Joseph Muenzer ꟾ Barbara K. Burton ꟾ Paul Harmatz ꟾ Luis González Gutiérrez-Solana ꟾ Matilde Ruiz‑Garcia ꟾ Simon A. Jones ꟾ Nathalie Guffon ꟾ Michal Inbar‑Feigenberg ꟾ Drago Bratkovic ꟾ Stewart Rust ꟾ Michael Hale ꟾ Yuna Wu ꟾ Karen S. Yee ꟾ David A.H. Whiteman ꟾ David Alexanderian

1. **TABLE S1** Idursulfase-IT treatment administration by intrathecal drug delivery device (D) or lumbar puncture (LP).
2. **TABLE S2** Summary of TEAEs in patients treated with idursulfase‑IT.
3. **TABLE S3** Idursulfase anti-drug antibody and neutralizing antibodies in serum and CSF.
4. **FIGURE S1** VABS II communication domain (A), daily living skills (B), socialization (C) and motor skills (D) scores by age in patients treated with idursulfase‑IT.
5. **FIGURE S2** Serum (A) and CSF (B) anti-idursulfase antibody titers in patients treated with idursulfase IT.

### TABLE S1 Idursulfase-IT treatment administration by intrathecal drug delivery device (D) or lumbar puncture (LP).

|  | **Week** | | | | | | | | | | | | | | | | | | | | | | | | | |  |  |  |  |
| --- | --- | --- | --- | --- | --- | --- | --- | --- | --- | --- | --- | --- | --- | --- | --- | --- | --- | --- | --- | --- | --- | --- | --- | --- | --- | --- | --- | --- | --- | --- |
|  | **4** | **8** | **12** | **16** | **20** | **24** | **28** | **32** | **36** | **40** | **44** | **48** | **52** | **56** | **60** | **64** | **68** | **72** | **76** | **80** | **84** | **88** | **92** | **96** | **100** | **104** |  |  |  |  |
| **Patient 1** | D | X | D | D | D | D | D | D | LP | D | D | D | D | D | D | D | X | LP | LP | LP | LP | LP | LP | D | D | D |  |  |  |  |
| **Patient 2** | D | LP | LP | LP | LP | LP | LP | X | LP | LP | LP | LP | LP | D | D | D | D | D | D | D | D | D | D | D | D | D |  |  |  |  |
| **Patient 3** | LP | LP | LP | LP | LP | LP | LP | LP | LP | LP | LP | LP | LP | LP | LP | LP | LP | LP | LP | LP | LP | LP | LP | LP | LP | LP |  |  |  |  |
| **Patient 4** | D | D | D | D | D | D | D | D | D | D | D | D | D | LP | LP | LP | LP | LP | LP | LP | LP | LP | LP | LP | D | D |  |  |  |  |
| **Patient 5** | D | D | D | D | D | D | D | D | D | D | D | D | D | D | D | D | D | D | D | D | D | D | D | D | D | LP |  |  |  |  |
| **Patient 6** | D | D | D | D | D | LP | D | D | D | D | D | LP | D | D | D | D | D | D | LP | D | D | LP | D | D | LP | D |  |  |  |  |
| **Patient 7** | D | D | D | D | D | D | D | D | D | D | D | D | D | D | D | D | D | D | D | D | D | D | D | D | D | D |  |  |  |  |
| **Patient 8** | D | D | D | D | D | D | D | D | D | D | D | D | D | D | D | D | D | D | D | D | D | D | D | D | D | D |  |  |  |  |
| **Patient 9** | D | D | D | D | D | D | D | D | D | D | D | D | D | D | D | D | D | D | D | D | D | D | D | D | LP | D |  |  |  |  |
|  | **Week** | | | | | | | | | | | | | | | | | | | | | | | | | | | | | |
|  | **108** | **112** | **116** | **120** | **124** | **128** | **132** | **136** | **140** | **144** | **148** | **152** | **156** | **160** | **164** | **168** | **172** | **176** | **180** | **184** | **188** | **192** | **196** | **200** | **204** | **208** | **212** | **216** | **220** | **224** |
| **Patient 1** | LP | LP | LP | X | D | LP | LP | LP | LP | D | D | LP | LP | LP | X | LP | LP | LP | LP | LP | LP | LP | LP |  |  |  |  |  |  |  |
| **Patient 2** | D | D | D | D | D | D | D | D | D | D | D | D | D | D | D | D | D | D | D | D | D | D | D |  |  |  |  |  |  |  |
| **Patient 3** | LP | LP | LP | LP | LP | LP | LP | LP | LP | LP | LP | LP | LP | LP | LP | LP | LP | LP | LP | LP |  |  |  |  |  |  |  |  |  |  |
| **Patient 4** | LP | LP | LP | LP | LP | LP | LP | LP | LP | LP | LP | LP | LP | LP | LP | LP | D | LP | LP | LP | LP |  |  |  |  |  |  |  |  |  |
| **Patient 5** | X | X | X | D | D | D | D | D | D | D | D | X | D | D | D | D | D | D | D | D | D | D | D | D | D | D | D | D | D | D |
| **Patient 6** | D | D | LP | D | D | D | LP | D | D | D | LP |  |  |  |  |  |  |  |  |  |  |  |  |  |  |  |  |  |  |  |
| **Patient 7** | D | D | D | D | D | D | D | D | D | D | D | D | D | D | D | D | D | D | D | D | D | LP | LP | D | D | D | D | D | D | D |
| **Patient 8** | D | D | D | D | LP | LP | LP | D | D | D | LP |  |  |  |  |  |  |  |  |  |  |  |  |  |  |  |  |  |  |  |
| **Patient 9** | X | D | D | D | D | D | D | D | D | D | LP |  |  |  |  |  |  |  |  |  |  |  |  |  |  |  |  |  |  |  |

D, drug delivery device; LP, lumbar puncture; X, no intrathecal dose administered.

### TABLE S2 Summary of TEAEs in patients treated with idursulfase‑IT.

| **Events** | **Substudy population (*N*= 9)** |
| --- | --- |
| At least one AE | 9 (100) |
| At least one severe AE | 3 (33.3) |
| At least one SAE | 8 (88.9) |
| At least one life-threatening AE | 0 |
| Discontinuation due to an AE | 0 |
| Deaths | 0 |
| At least one AE related to IV idursulfase infusion | 3 (33.3) |
| At least one AE related to idursulfase‑IT | 7 (77.8) |
| At least one IDDD surgical procedure-related AE | 7 (77.8) |
| At least one IDDD-related AE | 8 (88.9) |
| At least one IT administration process-related AE | 5 (55.6) |
| Associated with administration via IDDD | 3 (33.3) |
| Associated with administration via lumbar puncture | 4 (44.4) |
| Most common TEAEs (occurring in at least five patients), by preferred term | |
| Diarrhea | 8 (88.9) |
| Pyrexia | 8 (88.9) |
| Vomiting | 8 (88.9) |
| Ear infection | 6 (66.7) |
| Nasopharyngitis | 6 (66.7) |
| Procedural pain | 6 (66.7) |
| Rhinorrhea | 6 (66.7) |
| Cough | 5 (55.6) |
| Headache | 5 (55.6) |
| Increased eosinophil count | 5 (55.6) |

*Note:* Data are *n* (%) unless otherwise specified.
AE, adverse event; IDDD, IT drug delivery device; IT, intrathecal; IV, intravenous; SAE, serious AE; TEAE, treatment-emergent AE.

### TABLE S3 Idursulfase anti-drug antibody and neutralizing antibodies in serum and CSF.

|  | **Serum** | | | | | | | | | **CSF** | | | | | | | | |
| --- | --- | --- | --- | --- | --- | --- | --- | --- | --- | --- | --- | --- | --- | --- | --- | --- | --- | --- |
| **Patient** | **1** | **2** | **3** | **4** | **5** | **6** | **7** | **8** | **9** | **1** | **2** | **3** | **4** | **5** | **6** | **7** | **8** | **9** |
| **Screening** |  |  |  |  |  |  |  |  |  |  |  |  |  |  |  |  |  |  |
| ADA | 163840 | 655360 | 160 | Negative | 10240 | 40960 | 163840 | Negative | 163840 | 2560 | 5120 | Negative | Negative | 160 | 640 | 2560 | Negative | 1280 |
| NAb | 800 | 400 | Negative | – | 100 | 200 | 400 | – | 400 | Negative | Negative | – | – | Negative | Negative | Negative | – | Negative |
| **Week 4** |  |  |  |  |  |  |  |  |  |  |  |  |  |  |  |  |  |  |
| ADA | 163840 | 655360 | 40 | 160 | 2560 | 40960 | 163840 | 10 | 163840 | 1280 | 5120 | Negative | Negative | 80 | 2560 | 5120 | Negative | 2560 |
| NAb | 800 | 1600 | Negative | 200 | 200 | 400 | 1600 | Negative | 200 | Negative | Negative | – | – | 100 | 200 | 100 | – | 400 |
| **Week 16** |  |  |  |  |  |  |  |  |  |  |  |  |  |  |  |  |  |  |
| ADA | 655360 | 2621440 | 160 | 40 | 640 | 163840 | 655360 | Negative | 40960 | N/A | 655360 | Negative | Negative | N/A | 2560 | 2560 | Negative | 20 |
| NAb | 3200 | 6400 | Negative | 100 | 100 | 400 | 3200 | – | Negative | N/A | 200 | – | – | N/A | Negative | Negative | – | Negative |
| **Week 28** |  |  |  |  |  |  |  |  |  |  |  |  |  |  |  |  |  |  |
| ADA | 10485760 | 2621440 | 40 | 10 | 2560 | 163840 | 10 | Negative | 81920 | N/A | 163840 | Negative | Negative | N/A | 5120 | 5120 | Negative | N/A |
| NAb | 3200 | 3200 | Negative | 100 | 100 | 200 | 3200 | – | 100 | N/A | 200 | – | – | N/A | Negative | Negative | – | N/A |
| **Week 40** |  |  |  |  |  |  |  |  |  |  |  |  |  |  |  |  |  |  |
| ADA | 10485760 | 655360 | 40 | Negative | 160 | 163840 | 655360 | Negative | 40960 | N/A | 327680 | Negative | Negative | N/A | N/A | N/A | Negative | N/A |
| NAb | 1600 | 3200 | Negative | – | 100 | 400 | 800 | – | 200 | N/A | 200 | – | – | N/A | N/A | N/A | – | N/A |
| **Week 52** |  |  |  |  |  |  |  |  |  |  |  |  |  |  |  |  |  |  |
| ADA | 2621440 | 655360 | Negative | Negative | 160 | 163840 | N/A | Negative | 40960 | N/A | 327680 | N/A | Negative | Negative | 20480 | 40960 | Negative | 1280 |
| NAb | 3200 | 3200 | – | – | 200 | 400 | N/A | – | 200 | N/A | 200 | N/A | – | – | Negative | Negative | – | Negative |
| **Week 64** |  |  |  |  |  |  |  |  |  |  |  |  |  |  |  |  |  |  |
| ADA | N/A | 655360 | Negative | Negative | 160 | 40960 | 655360 | Negative | 10420 | N/A | 327680 | Negative | Negative | N/A | 10240 | 40960 | Negative | N/A |
| NAb | N/A | 1600 | – | – | Negative | 200 | 800 | – | 200 | N/A | Negative | – | – | N/A | Negative | Negative | – | N/A |
| **Week 76** |  |  |  |  |  |  |  |  |  |  |  |  |  |  |  |  |  |  |
| ADA | 2621440 | 655360 | 10 | Negative | 160 | 163840 | 655360 | Negative | 163840 | 81920 | 163840 | Negative | Negative | N/A | 5120 | N/A | Negative | N/A |
| NAb | 800 | 800 | 100 | – | Negative | 400 | 1600 | – | 400 | 200 | 100 | – | – | N/A | N/A | N/A | – | N/A |
| **Week 88** |  |  |  |  |  |  |  |  |  |  |  |  |  |  |  |  |  |  |
| ADA | 2621440 | 655360 | Negative | Negative | 40 | 40960 | 655360 | Negative | 40960 | 163840 | 163840 | Negative | Negative | N/A | 5120 | N/A | Negative | N/A |
| NAb | 800 | 1600 | – | – | 100 | 200 | 1600 | – | 400 | 200 | 200 | – | – | N/A | Negative | N/A | – | N/A |
| **Week 100** |  |  |  |  |  |  |  |  |  |  |  |  |  |  |  |  |  |  |
| ADA | 10485760 | 2621440 | Negative | Negative | Negative | 40960 | 655360 | Negative | 163840 | 655360 | 163840 | Negative | Negative | N/A | 10240 | 10240 | Negative | 5120 |
| NAb | 800 | 800 | – | – | – | 800 | 1600 | – | 400 | 400 | Negative | – | – | N/A | Negative | N/A | – | Negative |
| **Week 112** |  |  |  |  |  |  |  |  |  |  |  |  |  |  |  |  |  |  |
| ADA | 655360 | 163840 | Negative | Negative | N/A | 163840 | N/A | Negative | 40960 | 327680 | 163840 | Negative | Negative | N/A | N/A | N/A | N/A |  |
| NAb | 800 | 1600 | – | – | N/A | 400 | N/A | – | 400 | Negative | Negative | – | – | N/A | N/A | N/A | N/A |  |
| **Week 124** |  |  |  |  |  |  |  |  |  |  |  |  |  |  |  |  |  |  |
| ADA | 655360 | 163840 | Negative | Negative | 40 | 10240 | 655360 | Negative | 163840 | 81920 | 163840 | Negative | Negative | Negative | N/A | N/A | Negative |  |
| NAb | 1600 | 800 | – | – | 100 | 400 | 1600 | – | 800 | 100 | Negative | – | – | – | N/A | N/A | – |  |
| **Week 136** |  |  |  |  |  |  |  |  |  |  |  |  |  |  |  |  |  |  |
| ADA | 655360 | 163840 | Negative | Negative | 160 | 163840 | N/A | Negative | 163840 | 327680 | N/A | Negative | Negative | N/A | N/A | N/A | Negative |  |
| NAb | 800 | 800 | – | – | Negative | 400 | N/A | – | 800 | N/A | N/A | – | – | N/A | N/A | N/A | – |  |
| **Week 148** |  |  |  |  |  |  |  |  |  |  |  |  |  |  |  |  |  |  |
| ADA | 655360 | 163840 | Negative | Negative | 40 | 40960 | 655360 | Negative | 40960 | 327680 | 163840 | Negative | Negative | Negative | N/A | N/A | Negative |  |
| NAb | 1600 | 800 | – | – | Negative | 200 | 3200 | – | 800 | Negative | Negative | – | – | – | N/A | N/A | – |  |
| **Week 160** |  |  |  |  |  |  |  |  |  |  |  |  |  |  |  |  |  |  |
| ADA | 2621440 | 163840 | Negative | Negative | 40 |  | 655360 |  |  | 327680 | 40960 | Negative | Negative | Negative |  | N/A |  |  |
| Nab | 400 | 1600 | – | – | Negative |  | 1600 |  |  | Negative | Negative | – | – | – |  | N/A |  |  |
| **Week 172** |  |  |  |  |  |  |  |  |  |  |  |  |  |  |  |  |  |  |
| ADA | 655360 | 163840 | Negative | Negative | 40 |  | 655360 |  |  | 163840 | 81920 | Negative | Negative | Negative |  |  |  |  |
| Nab | 400 | 800 | – | – | Negative |  | 1600 |  |  | Negative | Negative | – | – | – |  |  |  |  |
| **Week 184** |  |  |  |  |  |  |  |  |  |  |  |  |  |  |  |  |  |  |
| ADA | 655360 | 163840 |  | Negative | 10 |  | 655360 |  |  | 327680 | 327680 |  | Negative | Negative |  |  |  |  |
| Nab | 800 | 800 |  | – | Negative |  | 3200 |  |  | Negative | Negative |  | – | – |  |  |  |  |
| **Week 196** |  |  |  |  |  |  |  |  |  |  |  |  |  |  |  |  |  |  |
| ADA |  |  |  |  | 10 |  | 163840 |  |  |  |  |  |  | Negative |  |  |  |  |
| NAb |  |  |  |  | Negative |  | 1600 |  | ` |  |  |  |  | – |  |  |  |  |
| **Week 208** |  |  |  |  |  |  |  |  |  |  |  |  |  |  |  |  |  |  |
| ADA |  |  |  |  | 10 |  | 163840 |  |  |  |  |  |  | Negative |  |  |  |  |
| NAb |  |  |  |  | Negative |  | 3200 |  | ` |  |  |  |  | – |  |  |  |  |
| **Week 220** |  |  |  |  |  |  |  |  |  |  |  |  |  |  |  |  |  |  |
| ADA |  |  |  |  | 40 |  | 655360 |  |  |  |  |  |  | Negative |  |  |  |  |
| NAb |  |  |  |  | Negative |  | 1600 |  | ` |  |  |  |  | – |  |  |  |  |

*Note:* Data are shown up to the data cut-off for the interim analysis (week 148 for all patients, and beyond week 148 for any patients for whom additional visits were completed before the cut-off).
ADA, anti-drug antibody; CSF, cerebrospinal fluid; N/A, not available; NAb, neutralizing antibodies.

### FIGURE S1 VABS II communication domain (A), daily living skills (B), socialization (C) and motor skills (D) scores by age in patients treated with idursulfase‑IT.


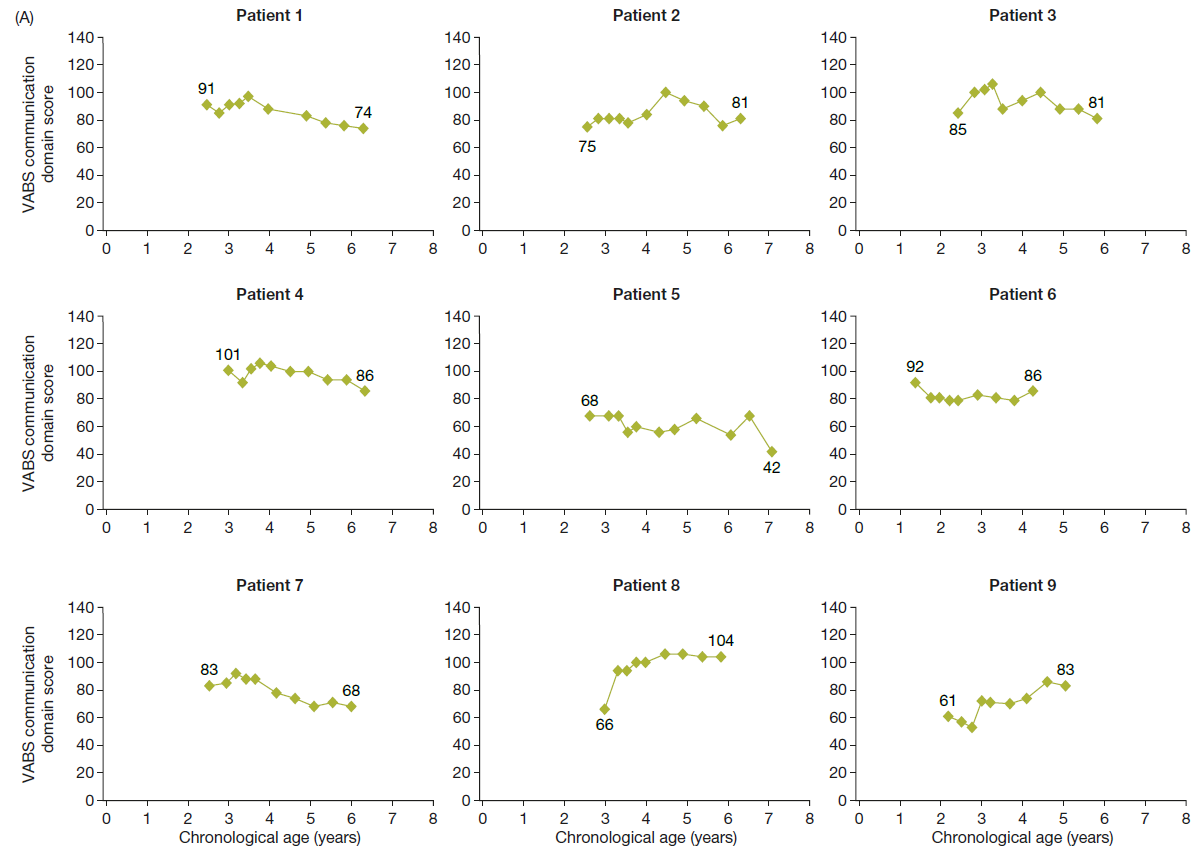


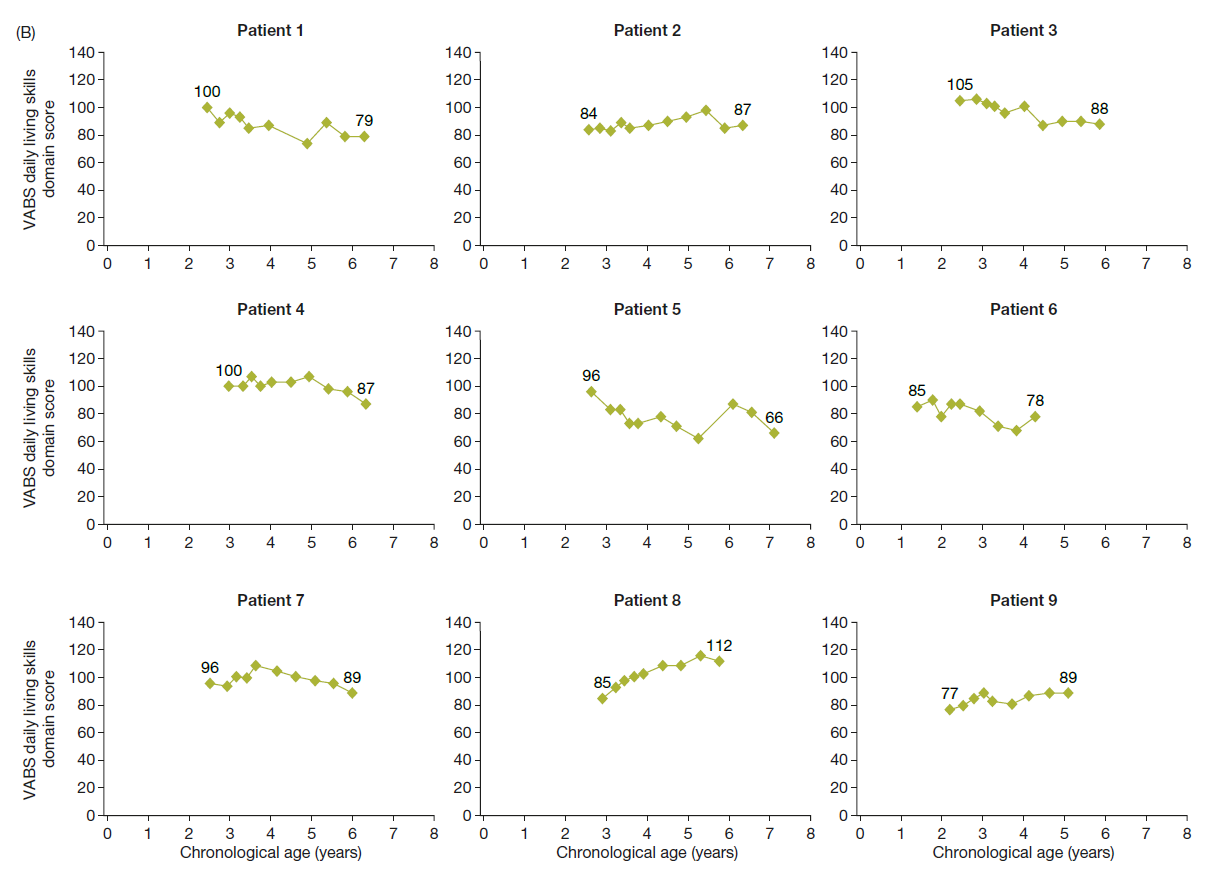


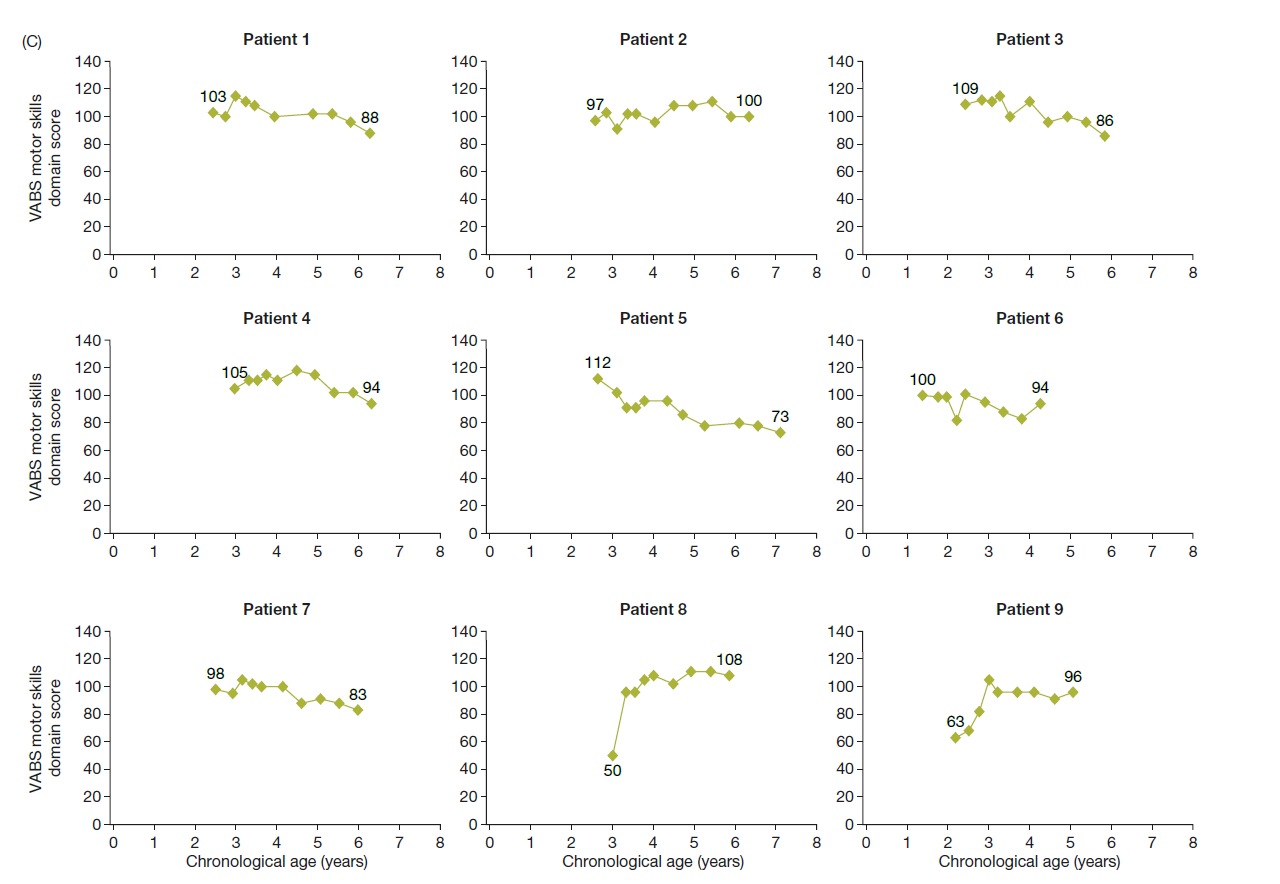


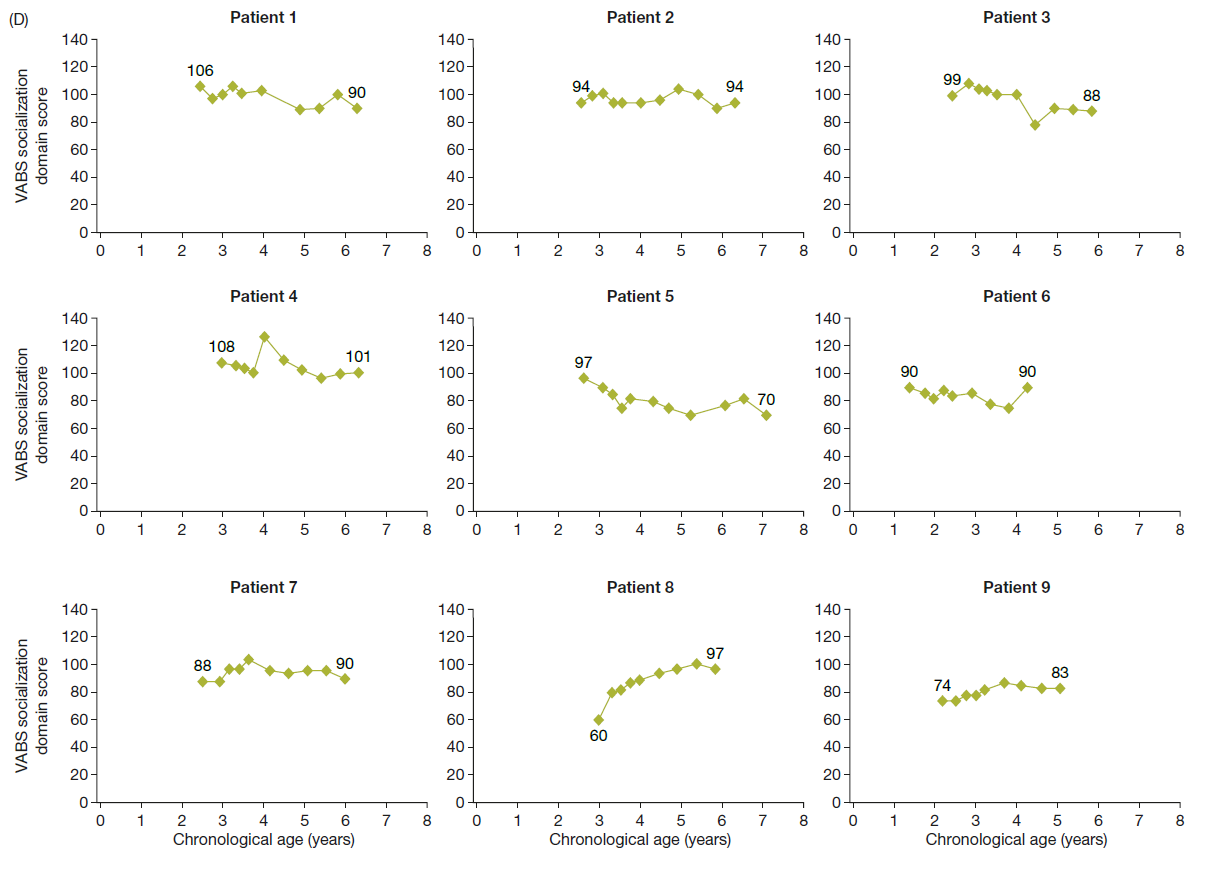


**FIGURE S2.** Serum (A) and CSF (B) anti-idursulfase antibody titers in patients treated with idursulfase IT

Antibody testing was conducted by a sponsor-designated contract research organization (Pharmaceutical Product Development, LLC, Richmond, VA, USA), using a bridging electrochemiluminescent immunoassay and the Meso Scale Discovery technology platform (Meso Scale Diagnostics, LLC, Rockville, MD, USA). For positive samples, neutralizing activity was detected by an I2S enzymatic activity assay using 4-methylumbelliferyl sulfate as substrate. CSF, cerebrospinal fluid; I2S, iduronate-2-sulfatase; IT, intrathecal.


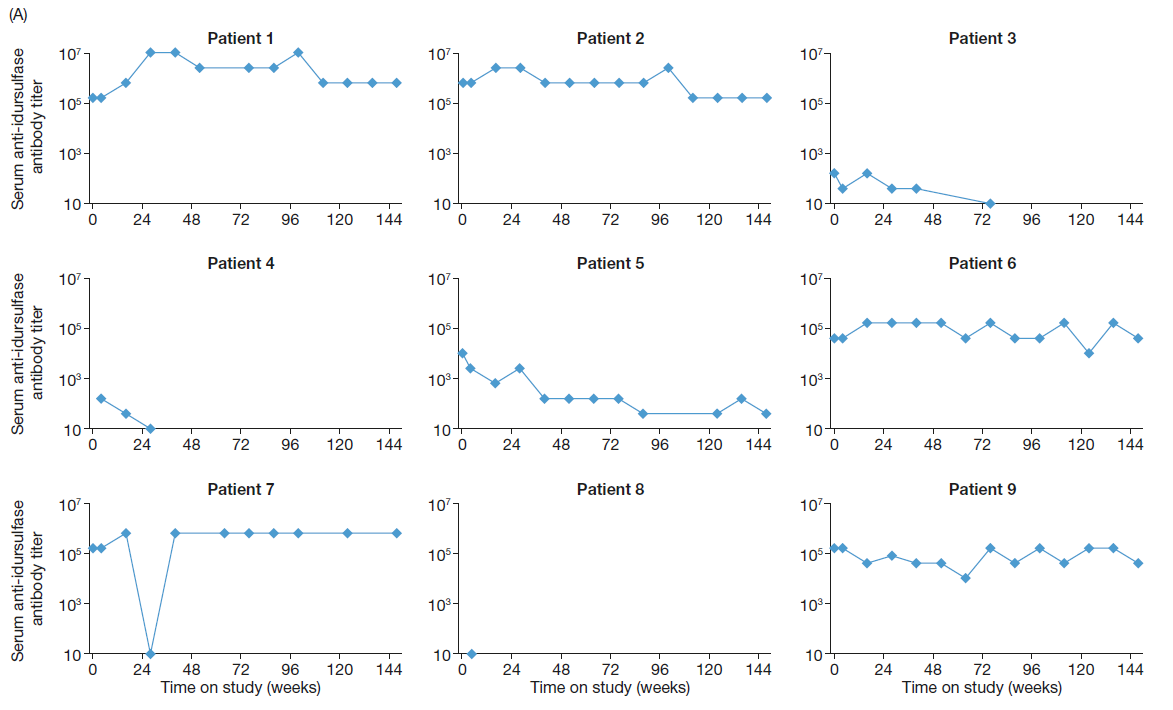


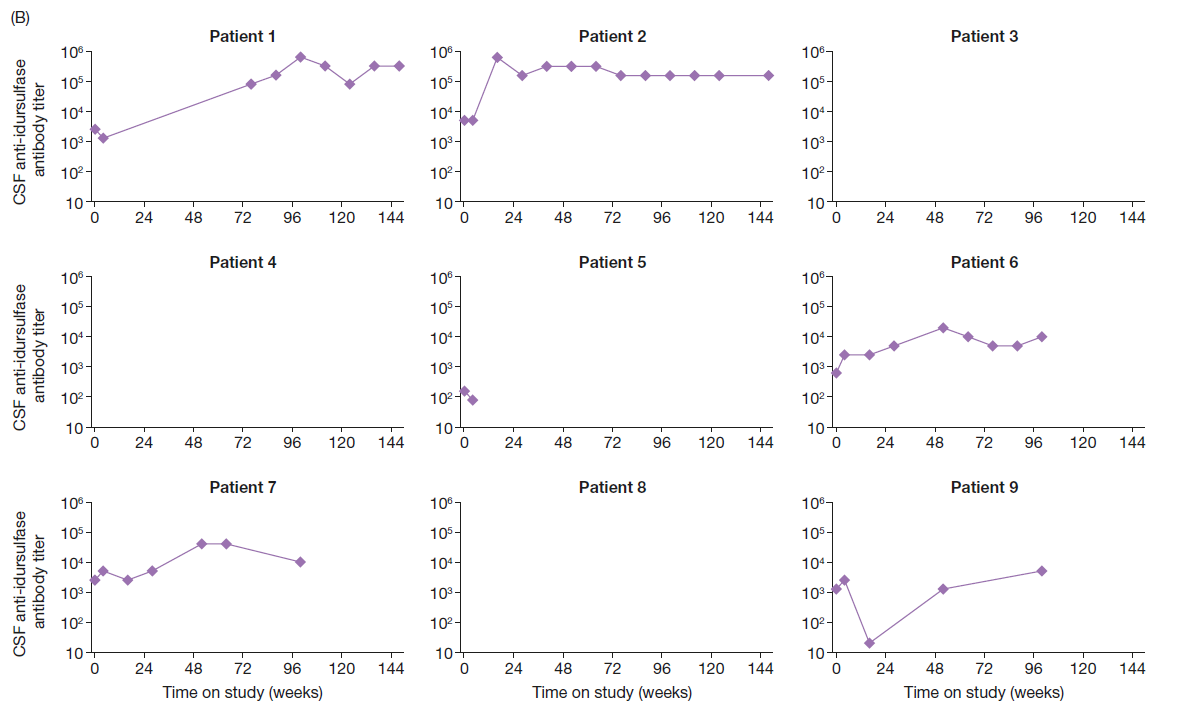

Supplement: Supplementary file 1 — Table S1. Idursulfase‐IT treatment administration by intrathecal drug delivery device (D) or lumbar puncture (LP). Table S2. Summary of TEAEs in patients treated with idursulfase‐IT. Table S3. Idursulfase anti‐drug antibody and neutralizing antibodies in serum and CSF. Figure S1. VABS‐II communication domain (A), daily living skills (B), socialization (C), and motor skills (D) scores by age in patients treated with idursulfase‐IT. Figure S2. Serum (A) and CSF (B) anti‐idursulfase antibody titers in patients treated with idursulfase‐IT. [file JMD2-67-e12443-s001.docx]
